# Combined effects of gabapentin with verapamil and metoprolol on cardiovascular function of anesthetized male rats

**DOI:** 10.1016/j.ejphar.2025.177828

**Published:** 2025-06-06

**Authors:** Sarah Pribil Pardun, Spencer Duff, Ian Papenfus, Will Bruening, Yulong Li, Gurudutt Pendyala, Lie Gao

**Affiliations:** a Department of Anesthesiology, University of Nebraska Medical Center, Omaha, NE, 68198, USA; b Department of Emergency Medicine, University of Nebraska Medical Center, Omaha, NE, 68198, USA

**Keywords:** Gabapentin, Verapamil, Metoprolol, Cardiovascular function, Rats

## Abstract

Gabapentin (GBP), at high plasma concentrations, negatively impacts cardiovascular function, causing bradycardia, hypotension, and impaired left ventricular (LV) function in rats. This study examines the combined effects of GBP with Verapamil (Ver) or Metoprolol (Met) on cardiovascular function in anesthetized male rats and its impact on Ca^2+^ current and calmodulin (CaM) protein expression in cultured H9c2 cells. Twenty-two male Sprague Dawley rats were assigned to four groups: Saline + Ver, GBP + Ver, Saline + Met, and GBP + Met. Under isoflurane anesthesia, rats underwent ECG monitoring and LV hemodynamic and blood pressure (BP) assessment. GBP (50 mg/kg) was administered intravenously, followed by Ver (0.05–0.4 mg/kg) or Met (0.5 mg/kg). In the first experiment, Ver induced a dose-dependent reduction in BP, heart rate (HR), and maximal dP/dt, while increasing minimal dP/dt, in both saline control and GBP-treated groups with the latter group showing greater effects. In the second experiment, GBP significantly reduced BP, HR, and maximal dP/dt while increasing minimal dP/dt compared to baseline. These effects were further exacerbated by Met, leading to greater reductions in BP, HR, and maximal dP/dt, and increases in minimal dP/dt compared to Met alone. In addition, in vitro experiments demonstrated that GBP reduced Ca^2+^ current and downregulated CaM protein. These findings suggest that, at high plasma concentrations, GBP enhances the negative chronotropic, negative inotropic, and hypotensive effects of both Ver and Met, further highlighting the need for additional preclinical and clinical studies to characterize the potential drug interactions between GBP and calcium channel blockers or beta-1 blockers, particularly in patients with cardiovascular disease.

## 1. Introduction

Gabapentin (GBP) is a widely used anticonvulsant primarily prescribed for the treatment of epilepsy, neuropathic pain, and off-label for various psychiatric conditions, including anxiety and bipolar disorder (Rose and Kam, 2002). Despite its efficacy in these domains, there has been growing concern regarding its potential adverse cardiovascular effects. Both clinical reports and preclinical studies suggest that GBP may induce significant alterations in cardiovascular function, including bradycardia and hypotension(Yasaei et al., 2025). Moreover, a recent study from our laboratory demonstrated that both acute (100 mg/kg) and chronic (50 mg/kg/day for 7 days) administration of GBP at high doses in rats resulted in negative chronotropic and inotropic effects, accompanied by hypotension and impaired left ventricular (LV) function (Pendyala et al., 2023). However, the interaction between GBP and commonly prescribed cardiovascular medications remains underexplored.

Patients with cardiovascular comorbidities, particularly those on calcium channel blockers (CCBs) and β1-adrenoceptor blockers, are of particular concern when prescribed GBP. Verapamil (Ver), a non-dihydropyridine CCB, is often used to treat hypertension, arrhythmias, and angina due to its ability to decrease myocardial contractility, heart rate (HR), and blood pressure (BP) through calcium channel blockade in cardiomyocytes, cardiac conduction system, and arterial smooth muscle (Elliott and Ram, 2011). Metoprolol (Met), a β1-selective adrenoceptor blocker, is commonly prescribed to manage hypertension, ischemic heart disease, and heart failure by reducing cardiac output through negative chronotropic and inotropic effects induced by blocking cardiac sympathetic effects(Wong et al., 2024). The concurrent use of GBP with Ver or Met raises concerns about potential greater depressant effects on the cardiovascular system than Ver or Met administration alone, given GBP’s demonstrated bradycardic and hypotensive effects.

Drug interactions between GBP and cardiovascular medications are clinically relevant, as their combined use may exacerbate the already significant cardiovascular depressant effects seen with Verapamil and Metoprolol. While some studies have suggested possible pharmacodynamic interaction of GBP with other drugs(Quintero, 2017), there is a dearth of experimental data on how GBP influences cardiovascular function when combined with these medications, especially in animal models where controlled investigations can be conducted.

In this study, we sought to investigate the combined effects of GBP with Ver and Met on cardiovascular function in anesthetized rats. Using direct hemodynamic measurements, we assessed changes in BP, HR, and LV function following the administration of GBP, either alone or in combination with Ver or Met. We hypothesize that GBP enhances the negative chronotropic, negative inotropic, and hypotensive effects of both Ver and Met. Given the widespread use of these medications in clinical practice, understanding their potential interactions with GBP is crucial to ensure safe therapeutic strategies for patients, particularly those with underlying cardiovascular conditions.

## 2. Materials and methods

### 2.1. Animal experiments

All animal procedures were conducted in accordance with the guidelines of the National Institutes of Health Guide for the Care and Use of Laboratory Animals and conformed to ARRIVE Guidelines (https://www.nc3rs.org.uk/arrive-guidelines, accessed on 9 September 2021), as approved by the Animal Care and Use Committee of the University of Nebraska Medical Center (UNMC-IACUC Protocols #24-012-00).

Twenty-two male Sprague Dawley rats (130–210 g) were divided into four groups to assess the effects of GBP on Ver- and Met-induced cardiovascular responses: Saline + Ver (n = 5), GBP + Ver (n = 7), Saline + Met (n = 5), and GBP + Met (n = 5). The rats received an intravenous bolus injection of GBP (50 mg/kg), followed by four incremental doses of Ver (0.05, 0.1, 0.2, and 0.4 mg/kg) or a single dose of Met (0.5 mg/kg). Saline (100–200 μL) was used as the vehicle control of GBP in the control rats to demonstrate the effects of Ver or Met alone.

Rats were anesthetized with 2 % isoflurane delivered in 100 % oxygen and placed on an electric heating pad to maintain body temperature at 37 °C throughout the experiment. For electrocardiogram monitoring, three micro-electrodes, made from platinum-iridium alloy wire (Pl 90 %, Ir 10 %, 0.1778 mm diameter), were implanted in the left and right chest walls and the lower left abdomen, serving as the positive, negative, and neutral leads, respectively. These electrodes were connected to an ECG amplifier (DP-311 Differential Amplifier, Warner Instruments). For arterial and LV hemodynamic measurements, a cathetertipped transducer (SPR-407, Millar Instruments, Houston, TX) was advanced through the right femoral artery into the abdominal aorta to measure BP and HR. Another catheter-tipped transducer (SPR-320NR, Millar Instruments, Houston, TX) was advanced via the right carotid artery into the left ventricular chamber to assess heart function. The amplified ECG and pressure transducer signals were input into a PowerLab^®^ data acquisition system (model 16S, AD-Instruments) with a sampling rate of 2 k/s. Data were monitored, recorded, and saved on a computer using LabChart^®^ software (version 8, AD-Instruments Ltd.) and analyzed using the ECG analysis module v8 and Blood Pressure Module software v1 (AD Instruments, Colorado Springs, CO), with an average of 50–100 cycles. A PE10 polyethylene tube was inserted into the right femoral vein for the administration of saline, GBP, Ver, and Met.

### 2.2. Cell experiments

To provide evidence showing direct effects of GBP on cardiomyocytes, we employed H9c2 (CRL-1446, ATCC, Manassas, VA), a cell line derived from embryonic BDIX rat myocardium(Witek et al., 2016), for investigating whether the electrophysiological characteristics and protein expression were changed by GBP. The cells were seeded in a 6-well plate at ~100,000 cells/well and grown in a complete media (CpM) (Dulbecco’s Modification of Eagle’s Medium (DMEM; Sig-ma-Aldrich) supplemented with 10 % fetal bovine serum (FBS; Gibco/ThermoFisher Scientific) and 1 % penicillin/streptomycin (P/S)), at 37 °C, 5 % CO_2_, 20 % O_2_, and 95 % humidity. The cells were allowed to adhere to the plate overnight. After which, the old media was replaced with fresh media containing GBP in 0.02, 0.2, and 2 mM. The cells were treated with the GBP media for 3 days. After GBP treatment, the cells underwent whole-cell patch-clamp recording to measure Ca^2+^ current (I_ca.L_) and western blot analysis to assess protein expression of Calmodulin (CaM).

### 2.3. Patch clamp recording

Resistance of the patch pipette was 4–6 MΩ when filled with pipette solution containing (in mM) 140 CsCl, 1 EGTA, 1 MgCl2, 5 Na2ATP, and 5 HEPES (pH 7.2, Tris). The bath solution containing (in mM): 135 tetraethylammonium (TEA)-Cl, 1.8 CaCl2, 2 MgCl2, 10 glucose, and 10 HEPES (pH 7.4, Tris). Nystatin (300 μg/ml) was included in the pipette solution for the perforated patch-clamp recordings. Cells were clamped at −80 mV and depolarized to 0 mV for 300 ms to evoke whole-cell I_ca.L_.

### 2.4. Western blot analysis

Protein was isolated using RIPA buffer (50 mM TrisHCl, 195 mM NaCl, 2 mM EDTA, 1 % NP-40, 0.1 % SDS) with 1 % protease inhibitor cocktail (ab65621 Abcam, Cambridge, UK) and then centrifuged at 20,000×*g* × 20 min at 4 °C. The protein concentration was measured using the Pierce bicinchoninic acid assay (Thermo Fisher Scientific, Waltham, MA, USA). An amount of 10 μg protein from each group was loaded separately onto a 10 % Bis-Tris wells (Invitrogen, Waltham, MA, USA) under reducing conditions, then transferred to a nitrocellulose membrane using iBlot2 (Invitrogen). Post transfer, the membranes were stained with Ponceau S stain (ThermoFisher Scientific, Waltham, MA, USA) to assess for equal protein loading detection and quantification. The membranes were treated on a rocker for 1 h at room temperature with 5 % fat free milk to block nonspecific antibody binding. After blocking, the membranes were incubated overnight at 4 °C with 1:1000 calmodulin (CaM) antibody (A4885; ABclonal, Woburn, MA, USA). The next day, the membranes were washed and treated with 1:2500 HRP conjugated anti-rabbit IgG for 1 h followed by additional phosphate-buffered saline-Tween 20 solution (PBST) washes. Blots were developed with 1:1 solution of Radiance Chemiluminescent Substrate and Luminol/Enhancer (Azure Biosystems, Dublin, CA, USA). G:BOX CHEMI XRQ imaging system (Syngene, Frederick, MD, USA) was used to visualize the blots, and the images acquired were quantified using the Image-J software version 1.52a.

### 2.5. Statistical analyses

Data are expressed as mean ± SD. Student’s t-test was used to compare differences between the two groups, using Prism 8 software. A P-value of <0.05 was considered statistically significant.

## 3. Results

### 3.1. Enhanced effect of GBP on Ver-induced cardiovascular inhibition

**Fig. 1.**
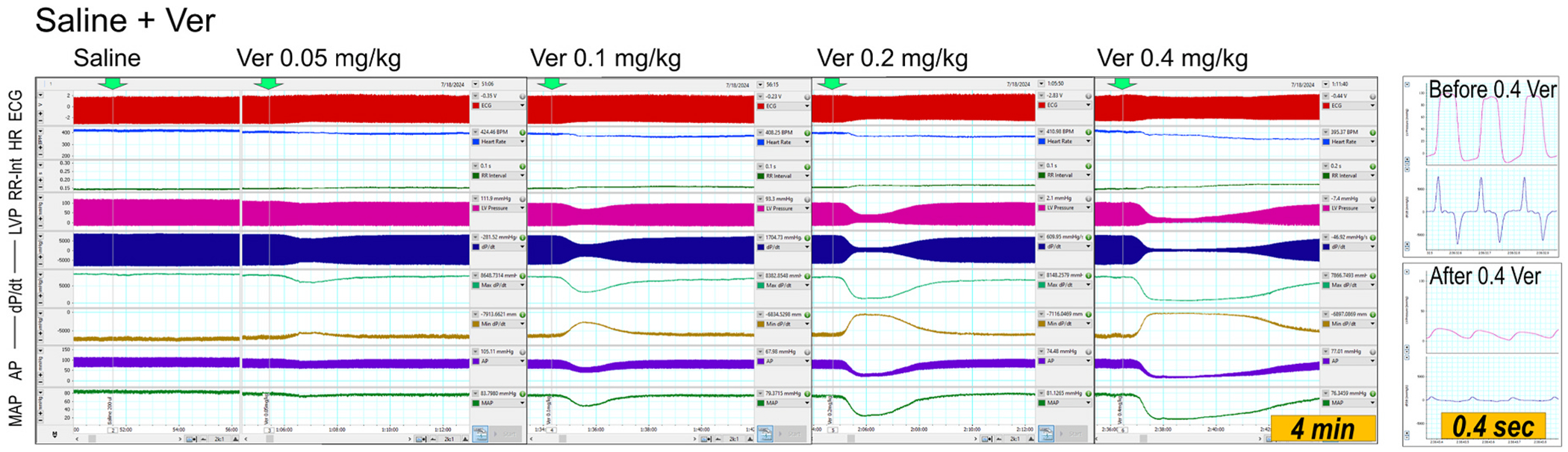
Representative recordings of Ver-induced cardiovascular inhibition in a dose-dependent manner.

Fig. 1 presents representative recordings illustrating the influence of Ver on cardiovascular function following saline pretreatment. As shown, saline had no effect on cardiovascular function, while Ver decreased dP/dt_max_, increased dP/dt_min_, and lowered BP in a dose-dependent manner, with the smallest effect observed at 0.05 mg/kg and the largest at 0.4 mg/kg. Ver exhibited negative chronotropic effects, which occurred after its negative inotropic effects. The rightmost panels display a 0.4-sec recording of LV pressure and dP/dt before (top panel) and after (bottom panel) treatment with Ver at a dose of 0.4 mg/kg. The dP/dt_max_ decreased from 7310.84 ± 661.24 to 961.57 ± 142.09 mmHg/s, while the dP/dt_min_ increased from −6413.08 ± 282.07 to −479.95 ± 107.31 mmHg/s (p < 0.0001, n = 5/group).

**Fig. 2.**
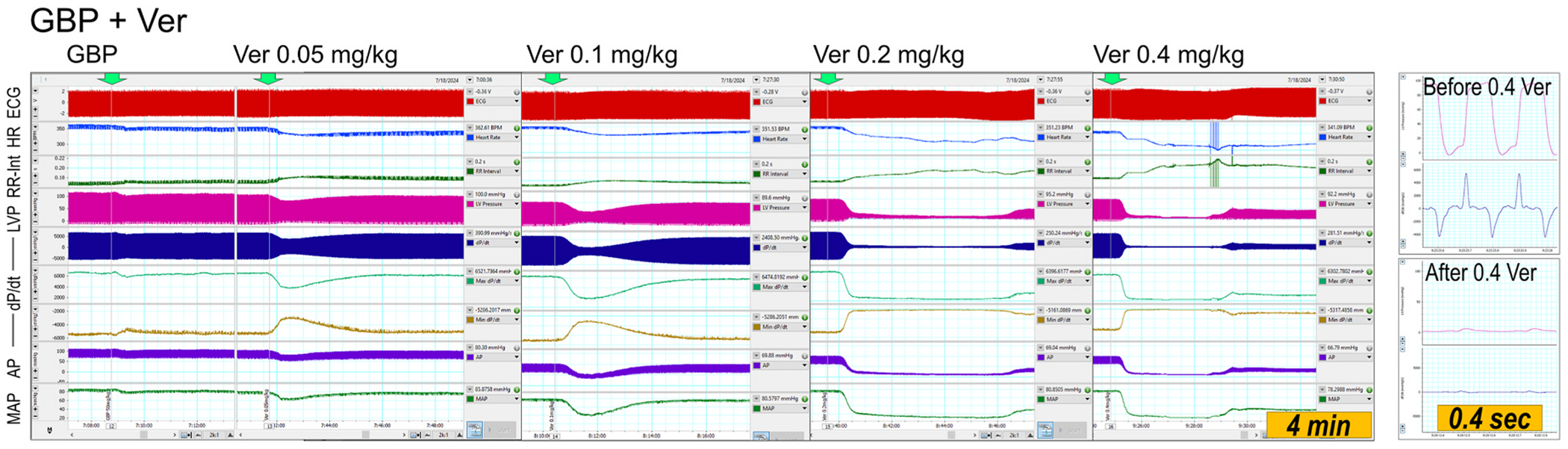
Representative recording of Ver-induced cardiovascular inhibition following GBP pretreatment.

Fig. 2 shows representative recordings of the effect of Ver on cardiovascular function following GBP pretreatment (50 mg/kg). As seen in this figure, GBP caused cardiovascular inhibition, and Ver further decreased HR, increased RR-interval, reduced dP/dt_max_, elevated dP/dt_min_, and lowered BP in a dose-dependent manner. The smallest effect was induced by 0.05 mg/kg, and the largest by 0.4 mg/kg. The rightmost panel shows a 0.4-sec recording of LV pressure and dP/dt before and after Ver injection at 0.4 mg/kg. The dP/dt_max_ decreased from 5771.46 ± 307.75 to 299.13 ± 134.13 mmHg/s, while dP/dt_min_ increased from −4997.81 ± 520.15 to −166.51 ± 50.88 mmHg/s (p < 0.0001, n = 7/group). On the other hand, IV saline following GBP did not evoke additional inhibitory effects on cardiovascular function (data not shown).

**Fig. 3.**
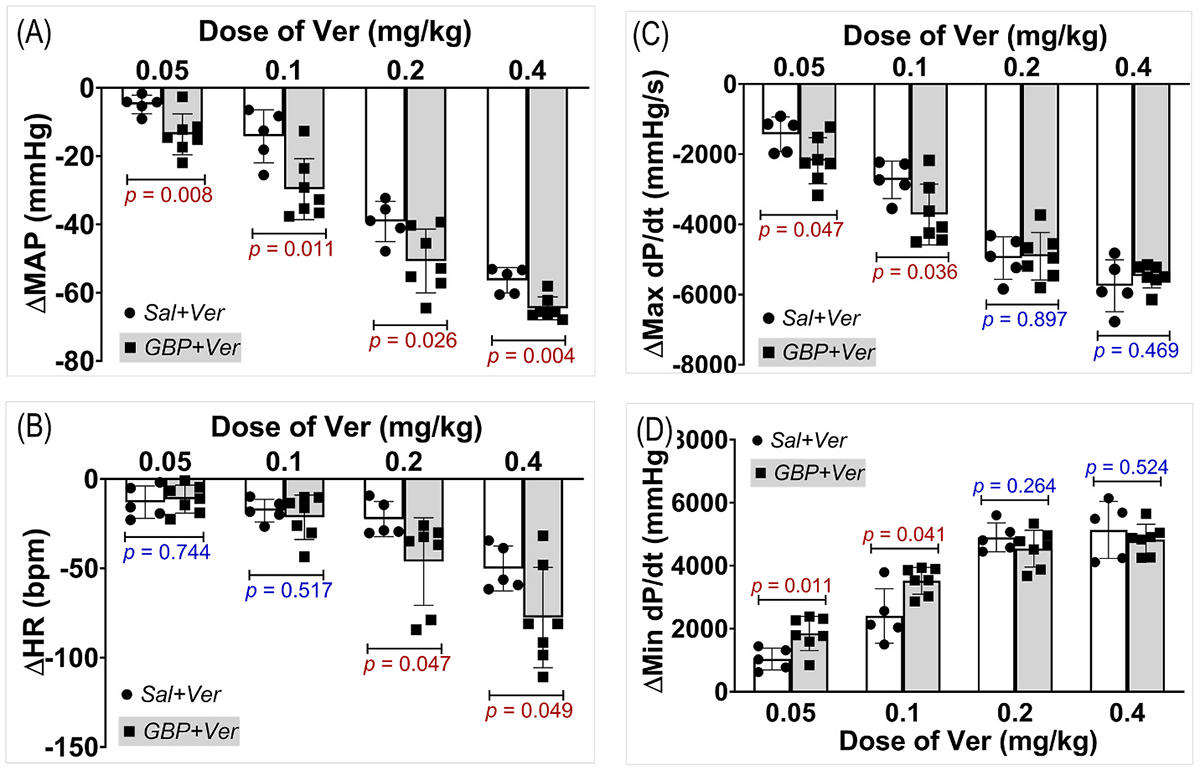
Group data show the changes of (A) MAP, (B) HR, (C) maximal dP/dt, and (D) minimal dP/dt induced by Ver following saline and GBP pretreatment. n = 5 and 7 in saline and GBP groups, separately.

Fig. 3 displays group data showing changes in MAP, HR, dP/dt_max_, and dP/dt_min_ induced by Ver after GBP pretreatment, with saline serving as the time course and vehicle control for GBP. Across all doses of Ver, GBP-pretreated rats experienced a significantly greater drop in BP than saline-pretreated rats. However, the GBP group showed significantly greater decrease in HR only at higher doses of Ver (0.2 and 0.4 mg/kg). The GBP group exhibited a significantly greater inhibition of LV function than the saline group only at lower doses. For example, after Ver treatment at 0.05 mg/kg, dP/dt_Min_ increased by 1035.63 ± 348.29 mmHg/s in the saline group and by 1850.14 ± 541.33 mmHg/s in the GBP group (*p* = 0.011; n = 5–7/group). Despite these changes in values, the absolute dP/dt_max_ and dP/dt_min_ values in GBP-pretreated rats receiving higher doses of Ver (0.2 and 0.4 mg/kg) were significantly lower and higher, separately, compared to those of saline-pretreated rats.

### 3.2. Enhanced inhibition of GBP and Met on cardiovascular function

**Fig. 4.**
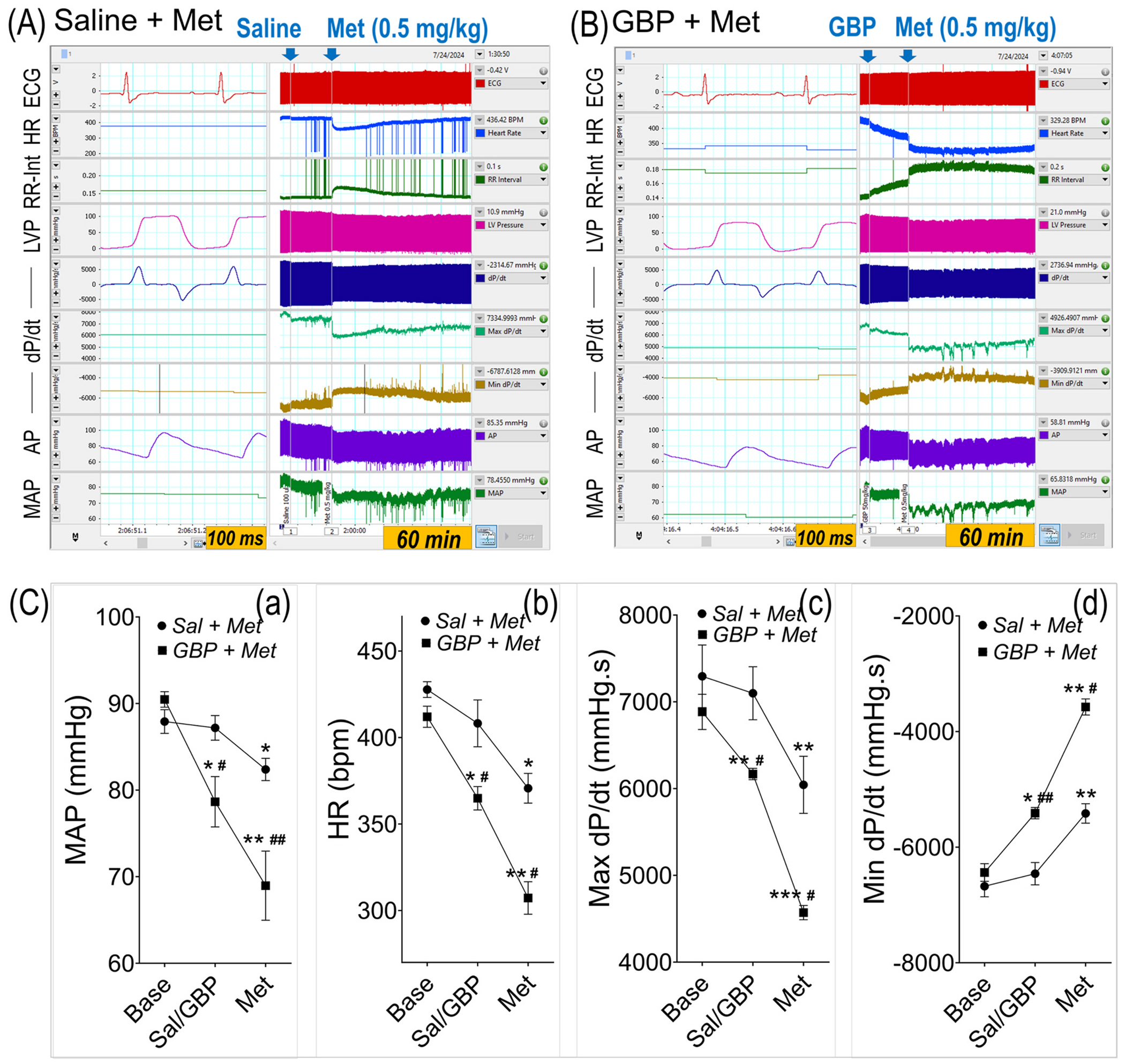
Effect of GBP on Met-induced cardiovascular inhibition. Top Panels: Representative recordings of Met-induced inhibition on cardiovascular function following (A) saline or (B) GBP pretreatment. Bottom Panels (C): Group data showing (a) MAP, (b) HR, (c) dP/dt_max_, and (d) dP/dt_min_ of the rats received Saline + Met or GBP + Met. *p < 0.05 and **p < 0.01 compared with baseline. ^#^p < 0.05 and ^##^p < 0.01 compared with Sal + Met group. n = 5/group.

Fig. 4 illustrates the effect of GBP pretreatment on Met-induced cardiovascular inhibition. Panels A and B show representative recordings of ECG, LV hemodynamics, and BP in rats treated with saline + Met or GBP + Met. Panel B presents group data for MAP, HR, dP/dt_max_, and dP/dt_min_ under baseline condition, after saline or GBP treatment, and after Met treatment. Since the effects of Met persist for over an hour, only one dose of Met (0.5 mg/kg) was tested in this experiment. It is evident that the reductions in MAP, HR, and dP/dt_max_, as well as the elevation in dP/dt_min_, were greater in the rats pretreated with GBP compared to the saline control group.

### 3.3. Effect of GBP on Ca^2+^ current in cardiomyocytes

**Fig. 5.**
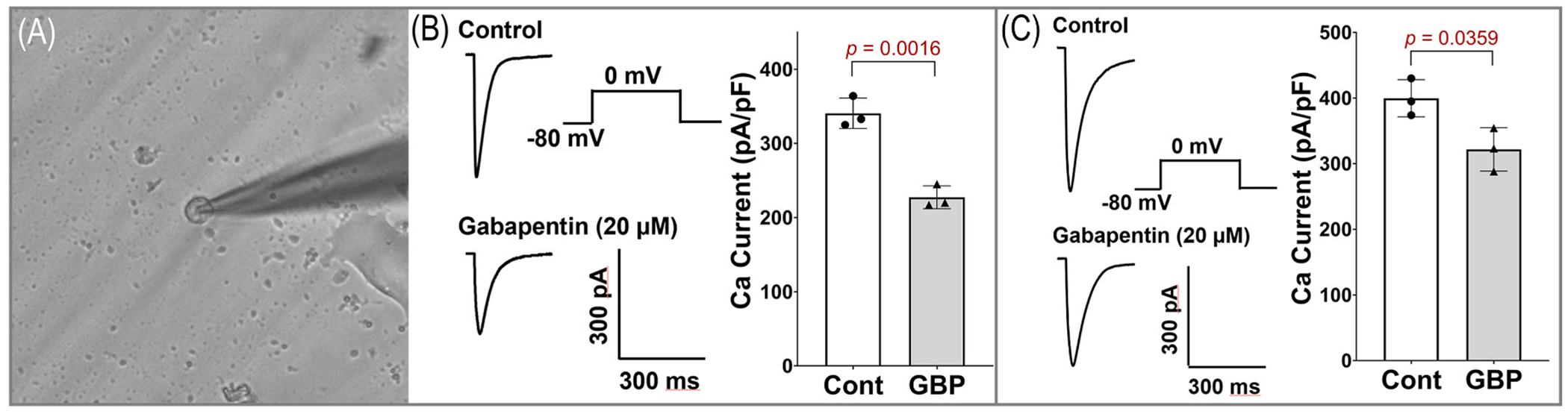
Whole cell patch clamp recording (A) shows reduced Ca^2+^ current in H9c2 by acute perfusion (B) or 3-day treatment with GBP (C) at 20 μM. Bar graph showing the mean data of I_Ca,L_ density (pA/pF) from the recordings of traces. Mean ± SD, n = 3 cells/group.

To determine whether GBP alters electrophysiological characteristics of cardiomyocytes, we employed whole cell patch-clamp technique with Axopatch 200B patch-clamp amplifier to record L-type calcium currents Ca^2+^ current (I_ca_) of the H9c2 cells, as shown in Fig. 5. These cells received an acute perfusion or 3-day chronic treatment with GBP at the concentration of 20 μM, a dose selected based on the publications of in vitro studies examining GBP’s effect on Ca^2+^ current and other electrophysiological parameters in vitro experiments of neurons and myotubes(Wamil and McLean, 1994; Alden and Garcia, 2002; Sutton et al., 2002). Panel A of Fig. 5 shows depiction of whole cell patch recording setup. A depolarizing single pulse (0 mV for 300 ms) was applied from a hold potential of −80 mV to record the inward ICa,L current. As can be seen in Panels B and C, both acute perfusion and 3-day treatment with 20 μM GBP significantly reduced the Ca^2+^ current.

### 3.4. Effect of GBP on calmodulin protein expression in cardiomyocytes

**Fig. 6.**
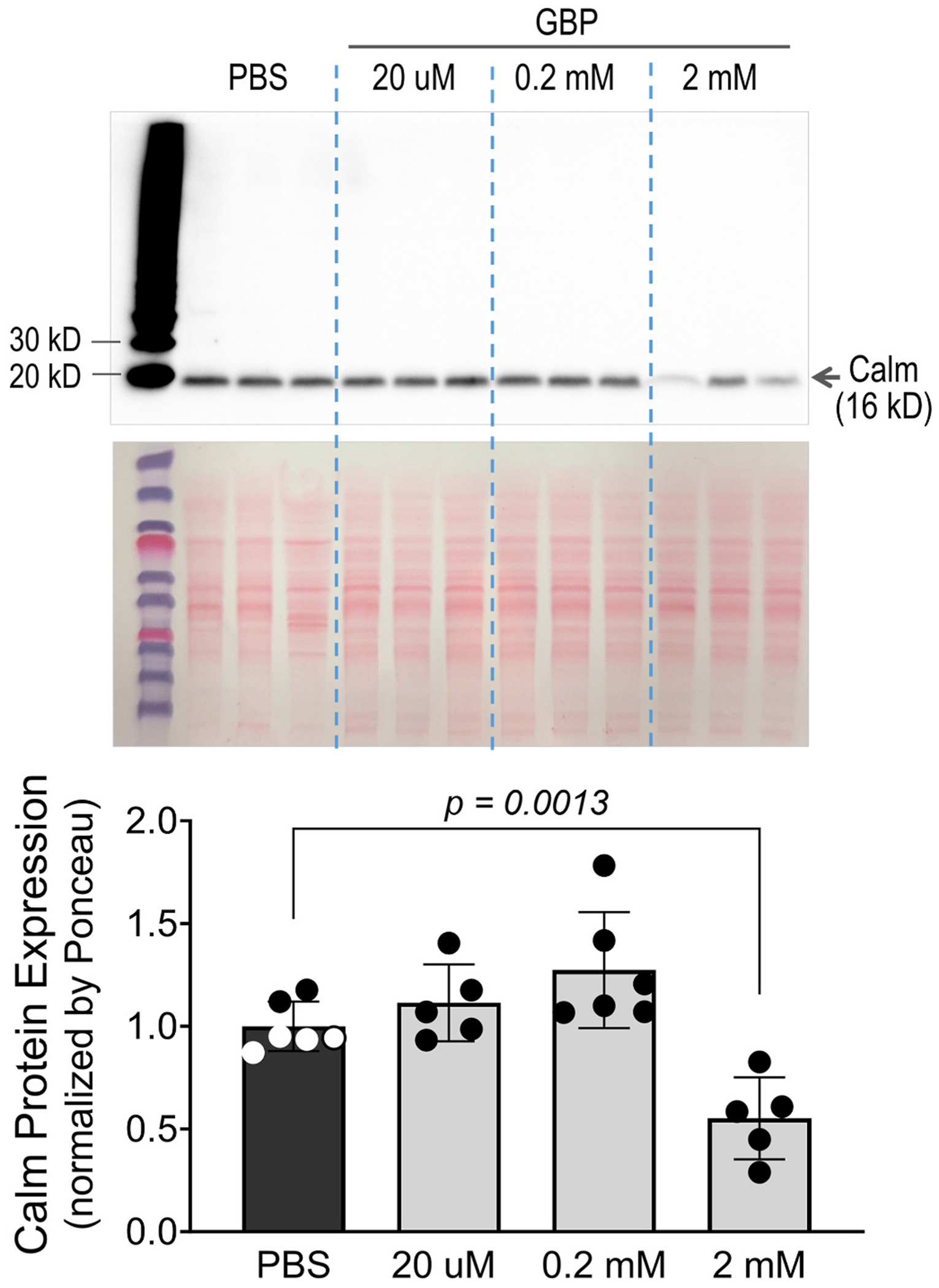
Western blot data show CaM protein expression. (A) original blots; (B) Bar graph showing the normalized CaM protein expression. Mean ± SD, n = 5–6/group.

To determine whether GBP alters expression of the protein associated with Ca^2+^ signaling pathway, we employed western blot to evaluate protein expression of CaM in the H9c2 treated with GBP at the concentrations of 0.02, 0.2, and 2 mM for 3 days. As can be seen in Fig. 6, GBP significantly down regulated CaM protein expression at the concentration of 2 mM, but not at 0.02 and 0.2 mM.

## 4. Discussion

The present study provides important insights into the combined effects of GBP with Ver and Met on cardiovascular function, particularly in terms of BP, HR, and LV function. Our findings suggest that, under the tested conditions, GBP may potentiate the cardiovascular depressant effect of Ver and Met. Although these findings are based on experiments in rats, they carry important clinical implications and underscore the need to investigate this potential interaction in human, particularly in patients undergoing concurrent treatment with GBP and cardiovascular medications.

### 4.1. GBP’s potentiation of Ver-induced cardiovascular depression

In Experiment 1, our data showed that Ver induced dose-dependent reductions in BP, HR, and LV function, as expected, due to its calcium channel blocking effects, which are known to reduce myocardial contractility and cause vasodilation(Eisenberg et al., 2004; Elliott and Ram, 2011). Importantly, rats pretreated with GBP exhibited an even greater reduction in BP across all doses of Ver, suggesting that GBP enhances the hypotensive effects of calcium channel blockers. This finding aligns with previous studies showing that GBP can cause hypotension on its own, likely by inhibiting sympathetic outflow(Behuliak et al., 2018). However, our data further demonstrates that GBP potentiates Ver-induced hypotension, a combination that could be particularly dangerous in patients already at risk for low blood pressure.

GBP and Ver exert enhanced inhibitory effects on cardiomyocytes by targeting different subunits of voltage-gated calcium channels, leading to enhanced negative chronotropic, negative inotropic, and hypotensive outcomes. GBP binds to the α2δ subunit, reducing the functional availability of calcium channels, leading to decreased Ca^2+^ influx. Ver directly binds to the α1 subunit and blocks the pore of calcium channels, reducing Ca^2+^ influx during the plateau phase of the action potential in cardiomyocytes. Together, they decrease intracellular calcium levels, which in pacemaker cells slows depolarization and reduces heart rate, and in cardiac myocytes, it weakens calcium-triggered muscle contraction, reducing the force of contraction. In vascular smooth muscle, this combined reduction in calcium influx causes vasodilation, lowering peripheral resistance and blood pressure. This complementary action on calcium channels amplifies their effects, enhancing the suppression of calcium-dependent processes in the heart and artery, leading to their potent cardiovascular effects.

With respect to HR, GBP potentiated Ver-induced bradycardia only at higher doses of Ver (0.2 and 0.4 mg/kg). This may reflect GBP’s own mild chronotropic effects, which become more pronounced when combined with the higher dose range of Ver, known to have a stronger negative chronotropic action(Nakaya et al., 1983). Additionally, GBP amplified Ver’s effects on LV function, with a significant inhibition of LV contractility, particularly in the lower Ver doses. The combination of these two drugs may thus result in a dangerous decrease in cardiac performance, particularly in patients with preexisting LV dysfunction.

### 4.2. GBP’s potentiation of Met-induced cardiovascular depression

In Experiment 2, Met, a selective β1-adrenoceptor blocker, also produced significant decreases in BP, HR, and LV function, as anticipated from its well-known inhibitory effects on cardiac output and myocardial contractility(Frishman, 2013). However, the administration of GBP before Met resulted in even more pronounced cardiovascular depression. Specifically, GBP pre-treatment significantly amplified the hypotensive, bradycardic, and negative inotropic effects of Met as indicated by the marked reductions in BP, HR, and maximal dP/dt, and increased minimal dP/dt.

The mechanisms by which GBP enhances the effects of Met may involve several factors. GBP is known to interact with voltage-gated calcium channels, which may affect sympathetic nervous system activity, contributing to reduced HR and BP(Chen et al., 2019). Additionally, GBP may impair baroreceptor reflex sensitivity, further compounding Met’s effects on cardiac function(Rabchevsky et al., 2011). These enhanced effects could pose significant clinical risks, particularly in patients with underlying heart failure or those taking multiple cardiovascular drugs.

### 4.3. GBP’s effects on CaM protein expression and Ca^2+^ current

As a well-known anticonvulsant and analgesic drug, GBP is widely recognized for its modulation of calcium channel activity, particularly through its high affinity for the α2δ subunit of voltage-gated calcium channels (VGCCs). Our experimental findings demonstrate that H9c2 cells incubated with GBP for three days, exhibit significantly reduced CaM protein expression and a concomitant decrease in Ca^2+^ current. These results align with the established mechanism of GBP action in altering intracellular calcium dynamics by inhibiting calcium influx. This reduction in Ca^2+^ current has profound implications for cellular functions dependent on calcium signaling, such as excitation-contraction coupling, gene transcription, and apoptosis regulation in cardiomyocytes. Previous studies have similarly shown that GBP modulates calcium influx, thereby potentially mitigating conditions like neuropathic pain and epileptic seizures(Taylor et al., 1998).

The reduction in CaM expression is particularly intriguing, as CaM is a pivotal calcium-binding messenger protein that regulates numerous calcium-dependent enzymes, ion channels, and signaling pathways. Its downregulation in response to GBP treatment suggests a broader modulation of calcium-mediated cellular processes. In cardiomyocytes, this could imply significant alterations in contractility and signal transduction pathways. Given that CaM plays a key role in regulating L-type calcium channels and ryanodine receptors, the observed decrease in its expression might exacerbate the reduction in Ca^2+^ current observed by patch clamp. This dual effect of GBP on both protein expression and ion flow could evoke an enhanced suppression on calcium-dependent signaling in H9c2 cells, offering insights into potential adverse effects in contexts of prolonged GBP exposure.

### 4.4. Clinical and public health implications

While our findings are based on preclinical models, they highlight a potential concern regarding the co-administration of GBP with cardiovascular medications such as Ver and Met. The significant potentiation of hypotension, bradycardia, and impaired left ventricular function observed in our study warrants further investigation to determine whether similar effects may occur in humans, particularly at clinically relevant drug concentrations. This is especially relevant in populations with preexisting cardiovascular conditions, where drug-induced hypotension and bradycardia could exacerbate symptoms of heart failure or lead to syncope, arrhythmias, or even cardiovascular failure.

Clinicians should consider dose adjustments of calcium channel blockers or β1-blockers when initiating GBP therapy. Moreover, careful monitoring of BP, HR, and cardiac function may be warranted in patients receiving these drug combinations. The results of this study also underscore the importance of future clinical investigations to evaluate the safety of these drug interactions in human subjects.

The choice of a 50 mg/kg IV dose for GBP in this study was based on several critical considerations aimed at ensuring adequate systemic exposure to investigate potential cardiovascular effects and pharmacodynamic interactions with Ver and Met. We acknowledge that this dose exceeds the minimum effective anticonvulsant threshold and is likely to yield plasma concentrations significantly higher than those typically observed with clinical therapeutic dosing in humans.

According to Welty et al. a 15 mg/kg IV dose of GBP in rats resulted in plasma concentrations of approximately 15.0 μg/mL at 0.25 h and 3.01 μg/mL at 4 h, levels consistent with the therapeutic anticonvulsant range(Welty et al., 1993). However, in preliminary experiments in our laboratory, this dose did not produce significant cardiovascular effects. In contrast, a 50 mg/kg IV dose elicited pronounced inhibition of left ventricular function, hypotension, and bradycardia in anesthetized rats according to our previous study(Pendyala et al., 2023).

Based on pharmacokinetic data reported by Vollmer et al. the expected peak plasma concentration following a 50 mg/kg IV dose of GBP in rats may approach ~100 μg/mL(Vollmer et al., 1986). While supratherapeutic, such concentrations allow for the exploration of dose-dependent off-target effects and potential toxicological profiles relevant for translational risk assessment. Indeed, it is important to consider the broader clinical and public health context regarding the GBP doses. Although GBP is primarily indicated for epilepsy, it is widely prescribed off-label for conditions such as anxiety, alcohol use disorder, and chronic pain, often at higher doses than those required for seizure control(Smith et al., 2016). Additionally, the misuse and abuse of GBP, including intravenous administration, have been increasingly reported, with cases involving total daily doses up to 12,000 mg(Lennox and Mangin, 2019). These patterns highlight the relevance of investigating the cardiovascular implications of supratherapeutic GBP exposure in a preclinical setting.

### 4.5. Limitations and future directions

While our study provides valuable insights, it is not without limitations. First, GBP has been reported to reduce the minimum alveolar concentration (MAC) of isoflurane(Boruta et al., 2012; Johnson et al., 2019; Chen et al., 2023). As a result, GBP-treated animals may have experienced comparatively deeper anesthesia at equivalent isoflurane concentrations, potentially leading to increased cardiovascular depression. This may confound the interpretation of GBP’s interactions with Ver and Met. Second, the dosing regimens used in this study may not accurately reflect clinical practices. Further research is needed to evaluate the effects of varying dosages and administration schedules. Third, this study focused solely on short-term outcomes. Chronic studies are necessary to assess the long-term effects of these drug interactions. Finally, the experiments were conducted exclusively on anesthetized male rats and lacked clinical data, which limits the generalizability of the findings. Future studies should include both sexes, conscious models, and clinical validation to enhance translational relevance.

### 4.6. Conclusion

**Fig. 7.**
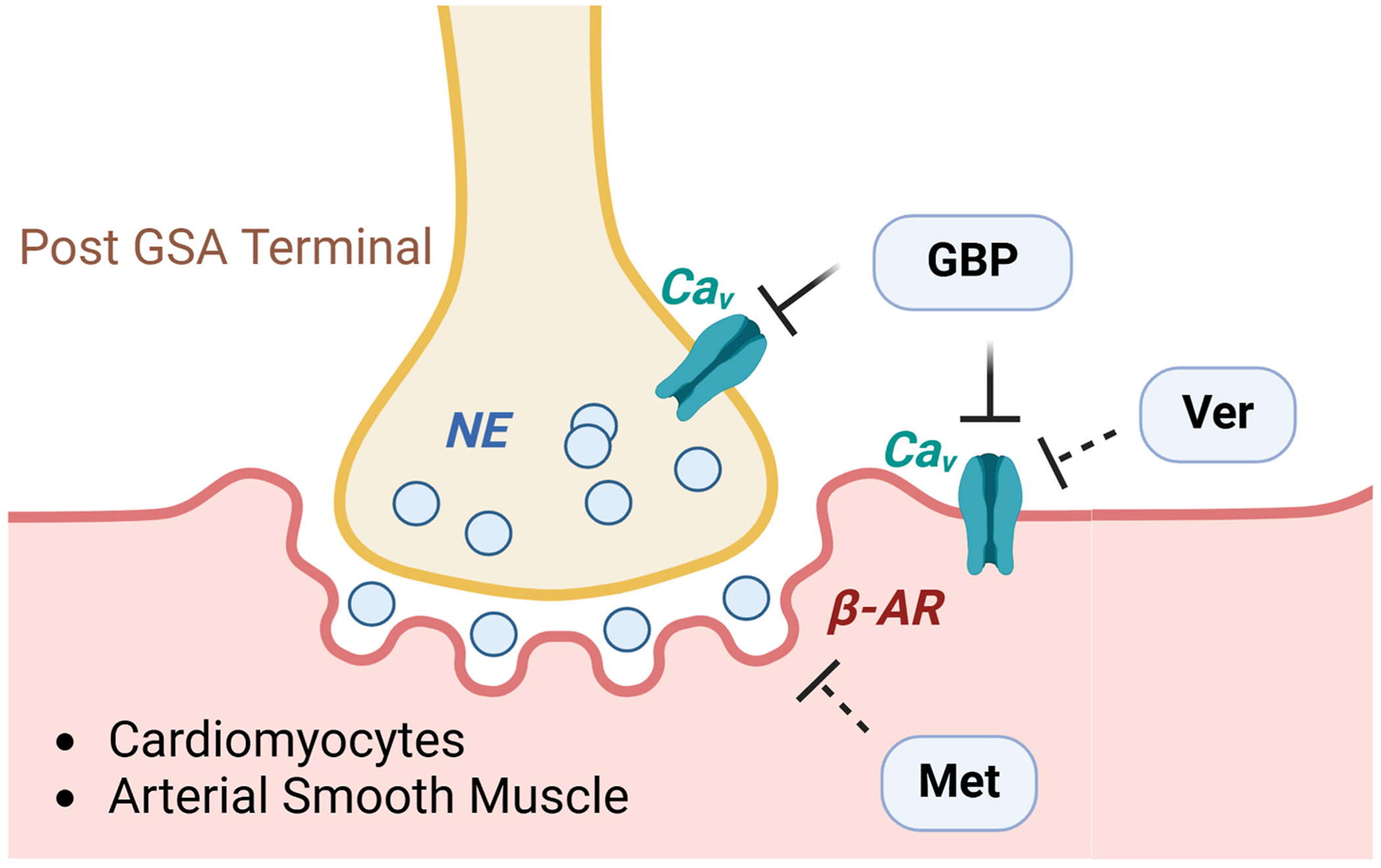
Graphical summary for combined effects of GBP with Ver and Met on cardiovascular system. GSA, ganglionic sympathetic axon; NE, norepinephrine; β-AR, β-adrenergic receptor.

In summary, this study demonstrates that GBP significantly potentiates the cardiovascular depressant effects induced by either Ver or Met in anesthetized rats, as summarized by Fig. 7. These findings suggest that co-administration of GBP with calcium channel blockers or β1-adrenoceptor blockers may require careful clinical management, including potential dose reductions and close monitoring of cardiovascular parameters. These findings raise questions about the potential long-term effects of gabapentin on cardiac function and highlight the need to evaluate its safety profile in patients with compromised cardiovascular systems. Understanding these mechanisms could open avenues for leveraging GBP’s effects in targeted therapies while mitigating potential risks.

## Data Availability

Data will be made available on request.
